# Cancer Registry follow‐up for 17 million person‐years of a nationwide maternity cohort

**DOI:** 10.1002/cam4.1222

**Published:** 2017-10-25

**Authors:** Matti Lehtinen, Heljä‐Marja Surcel, Kari Natunen, Eero Pukkala, Joakim Dillner

**Affiliations:** ^1^ University of Tampere Tampere Finland; ^2^ Karolinska Institute Stockholm Sweden; ^3^ European Science Infrastructure Services EEIG Stockholm Sweden; ^4^ National Institute for Health & Welfare Helsinki Finland; ^5^ Biobank Borealis of Northern Finland Oulu University Hospital Oulu Finland; ^6^ Finnish Cancer Registry Institute for Statistical and Epidemiological Cancer Research Helsinki Finland

**Keywords:** Biobank, cancer causes, cohort, epidemiology, tumor markers

## Abstract

Population‐based Finnish Maternity Cohort (FMC) comprises 2M first trimester sera collected from 1M women during 33 years. Informed consent is by the opt‐out principle, and linkages with cancer and population registries provide a base for over time and over generation studies. Follow‐up for 17M person‐years by the end of 2014 can identify 39,700 cases of invasive cancer and 18,900 cases of premalignant breast and cervix lesions, and basal cell carcinoma diagnosed after serum sampling. For women with multiple pregnancies, serial samples taken before cancer diagnosis are available. Offspring of the women have developed more than 4000 cancers. For 100,000 individuals, samples taken during the pregnancies of both their mothers and grandmothers enable familial cancer studies. FMC continues to collect samples, and surveillance of exposures or interventions like vaccination programs is feasible. In summary, the FMC is a unique, accessible biobank for epidemiological, biomarker, and surveillance studies on cancer.

## Introduction

In the hierarchy of epidemiological evidence on causes of cancer, longitudinal studies (either nested case–control or case–cohort studies) are second only to randomized trial evidence 1, 2. The Finnish Maternity Cohort (FMC) was established in 1983 to provide a resource for such studies. All women in Finland are at their 12th week of pregnancy offered screening for congenital infections (hepatitis B, HIV, and syphilis). The residual serum samples are stored in the FMC 3. There is a separate legal basis for the collection and scientific use of FMC (The law of the National Institute for Health and Welfare 668/2008). Since 2001, a nationwide informed consent system based on the opt‐out principle with negligible drop out has been in operation.

An example of pioneering cancer research based on the FMC are longitudinal studies documenting that exposure to human papillomavirus (HPV) types 16 and 18 causes an excess risk for later development of both cervical, other anogenital and oropharyngeal cancers 4, 5, 6. FMC's serial samples have been useful for disentangling the temporal order of events in carcinogenesis, for example, antagonistic interaction of different HPV types 7, and disclosing smoking as an independent risk factor of cervical cancer 8. The prediagnostic serial samples also enable studies on the sensitivity and specificity of biomarkers for future cancer diagnosis and screening 9. High‐quality data on the gestational date of the sample collection enable studies on hormones and cancer 10. The FMC is positioned as an international Open Access resource. Linkage of personal identifiers with the nationwide, population‐based Finnish Cancer Registry and other population‐based health and trial registries establishes a unique study base for longitudinal studies 11.

## Material and Methods

At the end of 2016 the FMC comprised 2.0 million serum samples from about 1 million women. As the congenital screening is repeated on each pregnancy, serial samples are available for about 50% of the women. Moreover, there are approximately 100,000 pairs of mothers and daughters, who both have donated sera to the FMC for scientific research.

We estimated the numbers of incident cancer cases in the FMC (Table 1). Cancer incidence in the FMC is similar to that in the general female population for all cancer types 11, with the exception of endometrial cancer which is decreased (43%) due to protection from pregnancy. Thus, it was possible to estimate the number of cancer cases by multiplying the person‐years generated by women in FMC by the end of year 2014 with cancer incidence rate in the Finnish female population in each 5‐year age category (www.cancerregistry.fi).

**Table 1:** Estimated^a^ numbers of incident invasive cancer cases in the Finnish Maternity Cohort 1983–2014

Primary site	ICD‐10 code	After first sample (*N* = 953,000)	After second sample (*N* = 604,000)	After third sample (*N* = 240,000)
All sites	C00–96, D32–33, D42–43, D45–47, D76	39,700	19,900	6800
Mouth, pharynx	C00–14	490	220	85
Lip	C00	10	5	1
Tongue	C02	110	50	5
Mouth, other	C03–06	110	50	15
Salivary glands	C07–08	110	50	20
Pharynx	C01, C09–14	130	55	20
Digestive organs	C15–26	3900	1900	700
Esophagus	C15	65	30	10
Stomach	C16	550	280	95
Small intestine	C17	110	55	20
Colon	C18	1400	700	250
Rectum, rectosigmoid, anus	C19–20	790	380	130
Liver	C22	135	50	20
Gallbladder, bile ducts	C23–24	140	60	20
Pancreas	C25	530	230	85
Other digestive organs	C26	30	10	0
Respiratory organs	C30–39	1050	420	130
Nose, sinuses	C30–31	35	15	5
Larynx, epiglottis	C32	15	5	2
Lung, trachea	C33–34	980	350	110
Mediastinum, pleura	C38	10	8	4
Bone	C40–41	90	40	10
Melanoma of the skin	C43	2200	1200	430
Skin, nonmelanoma	C44	400	180	60
Mesothelioma	C45	20	9	3
Autonomic nervous system	C47	10	4	0
Soft tissues	C48–49	280	140	60
Breast	C50	18,400	9200	3000
Female genital organs	C51–58	3800	1700	550
Cervix uteri	C53	1130	630	260
Corpus uteri	C54	1100	400	130
Uterus, other	C55, C58	20	5	1
Ovary	C56	1100	500	150
Other female genital	C51–52, C57	280	110	25
Placenta		15	4	2
Urinary organs	C64–68	900	400	160
Kidney	C64	620	290	120
Bladder and urinary tract	C65–68	270	110	40
Eye	C69	70	30	10
Brain, central nervous system	C70–72, D32–33, D42–43	2500	1400	500
Thyroid gland	C73	2300	1300	520
Other endocrine glands	C74–75	50	20	5
Ill defined or unknown	C76, C80	300	130	40
Lymphoid and hematopoietic tissue	C81–96, D45–47, D76	2600	1300	430
Hodgkin lymphoma	C81	350	180	55
Non‐Hodgkin lymphoma	C82–86, C96, D76	1200	600	200
Myeloma	C90	220	100	25
Leukemia	C91–95	600	300	90

a 96% of the Finnish pregnant women between 1983 and 2016 have consented to participate in the FMC. As the women can withdraw consent at will the numbers should be considered as estimates.

## Results

There are approximately 40,000 prospectively occurring cases of cancer within 17.2 million person‐years of follow‐up up to 2014. During the first 5 years after pregnancy, the number of new cancer cases is moderate but increases when the women become older. Breast cancer comprised almost half of all incident invasive cancer cases identified (18,400 cases, Table 1).

The next 10 most common cancer types were as follows: thyroid cancer (2300 cases), melanoma (2200 cases), colorectal cancers (2190 cases), lymphomas (1550 cases), cervical cancer (1130 cases), endometrial cancer (1100 cases), ovarian cancer (1100 cases), lung cancer (980 cases), kidney cancer (620 cases), and leukemia (600 cases). Noninvasive cancers include 10,000 cases of in situ cervical cancer, 7400 cases of basal cell carcinomas, and 1500 cases of in situ breast carcinomas.

The number of childhood cancer cases in the offspring of the FMC donors (4000 cases) is sizeable (Table 2). Especially, the numbers of childhood leukemias and lymphomas (altogether more than 1600 cases) are high (Table 2).

**Table 2:** Estimated^a^ numbers of incident invasive cancer cases in the offspring of the Finnish Maternity Cohort participants (FMC) 1983–2014

Cancer type	ICD10	Boys				Girls				All			
		0–4	5–9	1 ± 14	0–14	0–4	5–9	10–14	0–14	0–4	5–9	10–14	0–14
All sites	C00–96, D32–33, D42–43, D45–47, D76	1180	535	445	2160	1020	420	410	1850	2200	955	855	4010
Mouth, pharynx	C00–14	<5	<5	10	10	0	<5	5	10	<5	5	15	20
Digestive organs	C15–26	20	10	25	60	15	10	45	70	35	25	70	130
Respiratory organs	C30–39	10	<5	5	15	10	5	0	15	15	10	5	30
Female genital organs	C51–58					10	5	20	35	10	5	20	35
Male genital organs	C60–63	30	5	5	40					30	5	5	40
Urinary organs	C64–68	110	20	5	135	105	20	5	130	220	40	10	270
Melanoma of the skin	C43	5	5	15	20	0	5	10	15	5	10	20	35
Skin, nonmelanoma	C44	0	5	5	10	5	0	5	5	5	5	5	15
Eye	C69	55	5	5	65	45	5	5	50	105	5	5	115
Thyroid gland	C73	5	5	5	15	0	5	15	20	5	10	20	35
Other endocrine glands	C74–75	90	10	10	110	90	5	5	100	180	15	15	210
Bone	C40–41	5	15	25	45	15	10	25	50	15	20	55	90
Soft tissues	C48–49	40	20	15	75	45	20	10	75	90	35	30	155
Ill defined or unknown	C76, C80	5	5	5	15	5	5	5	15	10	10	5	25
Autonomic nervous system	C47	40	5	5	50	40	5	5	50	80	5	5	90
Brain, CNS	C70–72, D32–33, 42–43	265	185	140	590	195	145	115	455	460	330	260	1050
Lymphoid, hematopoetic tissue	C81–96, D45–47, D76	500	235	175	910	445	175	155	770	940	410	325	1675

a 96% of all Finnish pregnant women between 1983 and 2014 have consented to participate in the FMC. As the women can later withdraw consent at will the numbers should be considered as estimates.

## Discussion

Cancer incidence in the FMC is similar to that in the general female population for all cancer types 11, with the exception of endometrial cancer which is decreased (43%) due to protection from pregnancy. With only 20 women opting out in 2017, the population‐based nature of FMC remains virtually intact.

In addition to longitudinal studies on cancer etiology and screening studies, the resource is also potentially useful for studies on genetic cancer risk. However, the first trimester serum samples contain measurable amounts of fetal DNA, which needs to be controlled for in genetic studies 12. Because the FMC is nationwide and contains samples from 94% of all Finnish pregnant women 11, very large‐scale over‐generation studies can be designed by linking the identities of the mothers to their offspring and/or relatives 13. Due to the long history of the FMC, prospects for studying congenital causes of cancer in the offspring of the women in FMC are also good.

Ongoing FMC projects include studies of the possible role of the same microbiome, that is, similar serological signature for mothers and daughters with the same cancer for the same types of *Chlamydia trachomatis* 14, HPV 7, *Helicobacter pylori,* or *Streptococcus galolyticus* in familial cervical, colorectal, and stomach cancers, respectively.

The FMC sample collection can also support clinical trials and surveillance purposes, including cancer control. For example, we are currently comparing the long‐term stability of antibody responses induced by either quadrivalent or bivalent vaccines against HPV among women who participated as adolescents in large randomized albeit population‐based trials of these vaccines 15. By linking the clinical trial files to the FMC files, it has been possible to identify serum samples collected up to 14 years postvaccination. Evaluation of vaccine‐induced antibody response in yet‐to‐be‐identified breakthrough cases makes search for correlates of protective immunity feasible.

This cohort profile describes the basic characteristics of the FMC and provides some examples of its potential use. The FMC is also a role model in strict safe guarding of integrity and compliance with data protection laws. While linkages of course need to be performed with identifiable data, once the samples are retrieved they are pseudonymized and it is not possible to link the research data to an identifiable individual. To promote the use of the FMC as an international resource for epidemiological cancer research, an international nonprofit company (ESIS EEIG) specializing in assisting international researchers with the data and samples they need has been founded and is available to facilitate the formal and logistic process to obtain access to the samples and data that international cancer research may need (www.esis.fi).

## Conflicts of Interest

None declared**.**
